# Identification of Genomic Structural Variations in Xinjiang Brown Cattle by Deep Sequencing and Their Association with Body Conformation Traits

**DOI:** 10.3390/ijms26115234

**Published:** 2025-05-29

**Authors:** Dan Wang, Tao Zhang, Menghua Zhang, Qiuming Chen, Mengjie Yan, Shengchao Ma, Jiangkun Wang, Xiaoxue Zhang, Kailun Ma, Lei Xu, Xixia Huang

**Affiliations:** College of Animal Science, Xinjiang Agricultural University, Urumqi 830052, China; wangdan01100330@163.com (D.W.); z13319734240@163.com (T.Z.); zhangmenghua810@126.com (M.Z.); cqm19860612@126.com (Q.C.); y13095066028@163.com (M.Y.); shengchaomasicau@163.com (S.M.); xjnywk@163.com (J.W.); zhangxiaoxue0726@163.com (X.Z.); makailun0829@163.com (K.M.)

**Keywords:** structural variation, whole-genome resequencing, selective signals, body conformation, Xinjiang Brown cattle

## Abstract

Xinjiang Brown cattle is an elite dual-purpose breed (raised for dairy and beef) developed in China. To elucidate its genomic architecture, we conducted whole-genome resequencing of 169 Xinjiang Brown cattle, followed by structural variation (SV) detection and a genome-wide association study (GWAS). We identified 71,668 SVs, among which deletions were the most prevalent, followed by translocations, inversions, duplications, and insertions. We further identified 1286 high-frequency SVs involving 2016 protein-coding genes. Through functional enrichment analysis of these genes, we revealed associations of genetic variation at genomic positions near genes implicated in immune response and disease resistance (*NFKBIZ* and *PTPRT*), growth and development (*HDAC4* and *MEF2A*), and milk production (*TP63*, *FABP4*, and *MEF2A*). GWAS analysis of 31 body conformation traits revealed 58 SVs significantly associated with five traits (chest width, rear udder width, udder depth, rump width, and heel depth) at the genome-wide level. Additionally, nine candidate genes (*CLINT1*, *EBF1*, *PAM16*, *GRIP1*, *CFAP54*, *SLC22A16*, *DOK5*, *ETAA1*, and *IPMK*) were identified as potentially involved in the genetic regulation of body conformation traits. These findings provide novel insights for genetic improvement strategies and indicate that precision breeding could further enhance the production performance of this breed in the future.

## 1. Introduction

The study of genomic structural variation (SV) dates back to the early 20th century. In 1913, Sturtevant discovered chromosomal inversions in *Drosophila* (fruit flies) [1], and other researchers identified transposable element insertions in maize [2] and repetitive sequences in the *Drosophila* genome [3]. These early discoveries laid the foundation for studies into genomic SVs. The term “genomic structural variation” first appeared in 2004 and has been extensively studied in the human genome [4]. The 1000 Genomes Project was the first enterprise to systematically reveal SVs in the human genome and map their distribution across different populations [5]. Similar to single-nucleotide polymorphisms (SNPs), SVs are another important molecular marker that covers a broader genomic region [6]. In regulatory regions, SVs may have a stronger effect on gene regulation than SNPs, as they can alter large sequences of nucleotides in the genome. A single SV can substantially modify all regulatory elements via insertion or deletion [7]. Moreover, SVs affect much larger genomic regions than single-nucleotide variations and short insertions/deletions [8]. When SVs occur in a gene, they can influence gene expression regulation and transcription–translation through various mechanisms; for example, they can disrupt gene function, alter gene dosage, and expose recessive alleles [9]. Genomic SVs typically arise during cell division or DNA repair and serve as crucial mechanisms for maintaining genetic diversity and adapting to environmental changes [10]. Accurately detecting SVs across the whole genome is essential for understanding their formation mechanisms and their effects on economically important traits.

SVs are closely associated with animal evolution, adaptability, and key economic traits. Using whole-genome sequencing data from 14 wild and 65 domestic yaks, Zhang et al. identified 2634 copy number variation (CNV) regions, revealing that CNVs may have played crucial roles in phenotypic changes during yak domestication and adaptation to high-altitude environments [11]. Additionally, SVs influence milk yield, milk composition, reproduction, and body conformation in cattle [12]. SNP genotyping and next-generation sequencing data from 1717 Nellore cattle identified 688 CNV regions overlapping with 286 quantitative trait loci (QTL) associated with important production traits, including 34 CNV regions related to growth traits in beef cattle [13]. Genotyping of 1116 Swiss Brown cattle using a high-density SNP array identified CNVs with the SVS 8.4.4 software (Golden Helix Inc., Bozeman, MT, USA). They identified nine CNVs significantly correlated with milk composition traits, five of which were located near candidate genes related to milk quality, such as *DGAT1*, *ABCG2*, and *GHR* [14]. A GWAS on CNVs and milk production traits in 26,362 Holstein bulls and cows identified 34 CNVs significantly associated with milk production, most of which overlapped with known QTL, confirming the correlation between CNVs and milk yield [15]. An SV in the regulatory region of *GALNTL6* was detected and found to be associated with feed efficiency and growth traits in cattle [16]. Whole-genome sequencing of samples representing Bos taurus identified 1265 CNV regions, with comparative analysis across beef cattle breeds revealing that several QTL related to growth and meat quality were located within CNV regions [17]. Overall, as a key form of genomic genetic variation, SVs provide valuable insights into phenotypic differences and improve our understanding of the relationship between genes and phenotypes. With further research, SVs may provide unique perspectives on animal genetic diversity in the future.

Xinjiang Brown cattle is the first dual-purpose breed (raised for dairy and beef) that was independently developed in China. Its tolerance to cold, tolerance to rough feeding, strong adaptability, and high-quality milk and meat make it highly favored on the market. Our previous studies identified SNPs and insertion–deletions associated with economically important traits in the Xinjiang Brown cattle population. For example, Zhou et al. conducted a GWAS and identified four candidate genes associated with milk production traits, including *CDH2*, which is associated with milk fat percentage, and *GABRB2*, which is associated with milk protein yield [18]. Similarly, Ju et al. analyzed 388 Xinjiang Brown cattle and discovered 13 insertion–deletion loci within the *FHIT* gene, 8 of which were significantly associated with milk fat percentage, milk protein percentage, and somatic cell score, which are key traits affecting milk production [19]. Furthermore, previous studies have assessed milk production traits in Xinjiang Brown cattle using SNP-based genetic evaluation [20]. These findings provide essential theoretical support for the genetic improvement of this breed.

In this study, we performed whole-genome resequencing of the Xinjiang Brown cattle genome using the DNBSEQ-T7 (MGI Tech Co., Ltd., Shenzhen, Guangdong, China) sequencing platform to comprehensively analyze the distribution characteristics of SVs and construct an SV map representing the breed. By annotating high-frequency SVs, we provide a reliable reference for the fine mapping of genes associated with economically important traits. Additionally, based on the identified SV data, we performed selection signal analysis and GWAS using SVs as molecular markers to explore their genetic associations with body conformation traits. Overall, this study provides new perspectives and theoretical support for understanding the genetic foundation and unique characteristics of Xinjiang Brown cattle.

## 2. Results

### 2.1. Genome Data Description

We performed whole-genome resequencing on 169 Xinjiang Brown cattle via high-throughput sequencing technology, generating over 10 TB of raw sequencing data. Following rigorous quality control procedures, we retained 74,514,505,005 clean reads, covering 10,287.95 GB. Statistical analysis of the BAM files using Qualimap v2.2.1 showed that the mapping rate of clean reads to the reference genome (ARS-UCD1.2 Btau_5.0.1Y) was 99.88%, with an average sequencing depth of 23.70×. Supplementary Table S1 provides detailed sequencing data statistics.

### 2.2. Detection of Genomic Structural Variations

**Figure 1:**
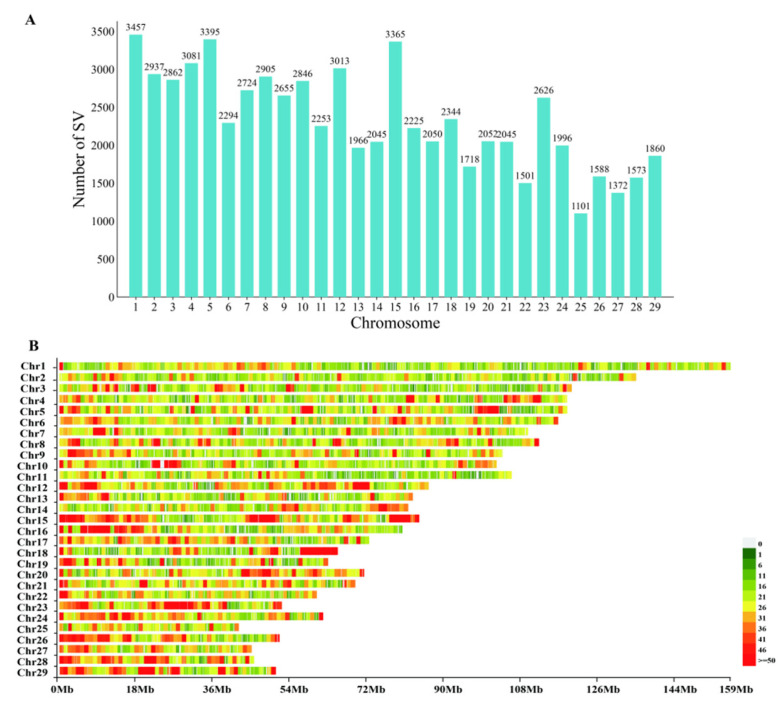
Distribution of SVs across chromosomes in Xinjiang Brown cattle. (**A**) Chromosomal distribution of SVs. (**B**) Structural variation map.

By integrating Manta v1.6.0, LUMPY v0.3.1, and Delly v1.1.6 for SV detection, followed by SURVIVOR filtering and merging, we obtained a high-quality SV dataset for Xinjiang Brown cattle and identified 67,849 SVs. Chromosomal distribution analysis revealed differences in the distribution of SVs across chromosomes: chromosome 1 harbored the largest number of SVs (3457), trailed by chromosomes 5 and 15. Conversely, chromosome 25 had the fewest SVs (1101). Figure 1 shows the SV distribution across chromosomes.

### 2.3. Characteristics of Structural Variations

**Figure 2:**
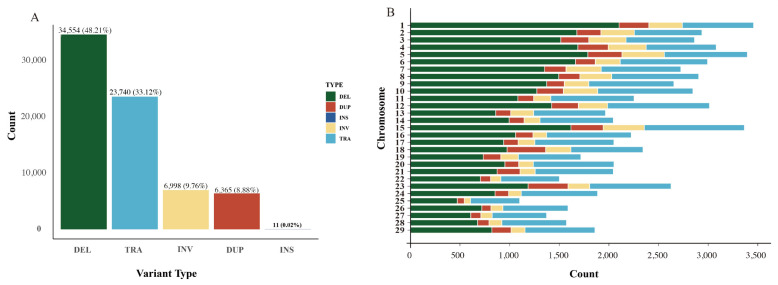
Proportions and chromosomal distribution of different SV types. (**A**) SV types; (**B**) Distribution of different SV types across chromosomes.

Among the five types of SVs, deletions were the most abundant, with a total of 34,554, constituting 48.21% of all variations. We show a 76-bp deletion on chromosome 25 (chr25:3,507,270–3,507,346) (Figure S1). Translocations were the second most common type of variation, with 23,740 occurrences, representing 33.12% of all variations. Insertions were the least frequent, with only 11 identified. Inversions and duplications had relatively similar proportions, representing 9.76% and 8.88%, respectively (Figure 2A). Regarding chromosomal distribution, deletions, duplications, inversions, and translocations were most prevalent on chromosomes 1, 5, 12, and 23, respectively, whereas insertions were distributed across only eight chromosomes (Figure 2B). Furthermore, length distribution analysis demonstrated that deletion sizes were predominantly in the 0–10 kb range, whereas inversion and translocation sizes were predominantly in the 100 bp–1 Mb range.

### 2.4. Annotation and Enrichment Analysis of High-Frequency SVs in Xinjiang Brown Cattle

Variants were retained if the allele frequency was >0.9. We identified 1286 high-frequency SVs, which were predominantly located in intergenic regions (825 SVs, 64.15%) and intronic regions (400 SVs, 31.10%), while only 32 SVs (2.49%) were found within exonic regions.

**Figure 3:**
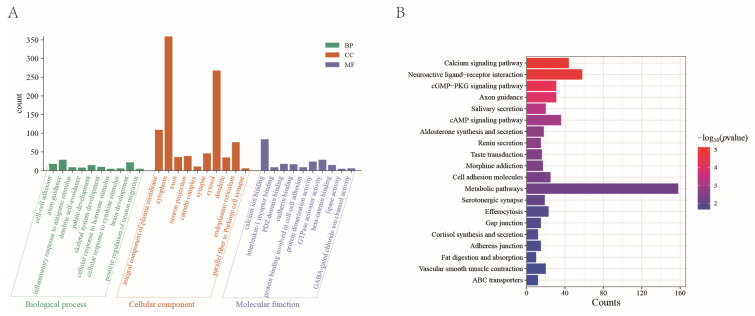
(**A**) GO and (**B**) KEGG enrichment analysis of genes in high-frequency SV regions of Xinjiang Brown cattle.

To investigate the biological significance of these high-frequency SVs, we performed gene annotation using ANNOVAR (v20230607) and identified 2016 protein-coding genes. Subsequently, we performed GO enrichment and KEGG pathway enrichment analyses. Figure 3A,B show the top 30 significantly enriched GO terms and the top 20 significantly enriched KEGG pathways, respectively.

The GO enrichment analysis identified 157 significant GO terms, including those associated with growth and development, such as skeletal system, kidney, and palate development. Additionally, immune-related terms, such as inflammatory response to antigenic stimuli and positive regulation of interleukin-2 production, were significantly enriched. Furthermore, terms related to neural function, including brain development, neuron differentiation, and synapse formation, were enriched. The KEGG enrichment analysis revealed 31 significantly enriched pathways that play potential roles in cell signaling, cellular interactions, and physiological function regulation.

### 2.5. Genome-Wide Association Analysis of Body Conformation Traits in Xinjiang Brown Cattle

**Figure 4:**
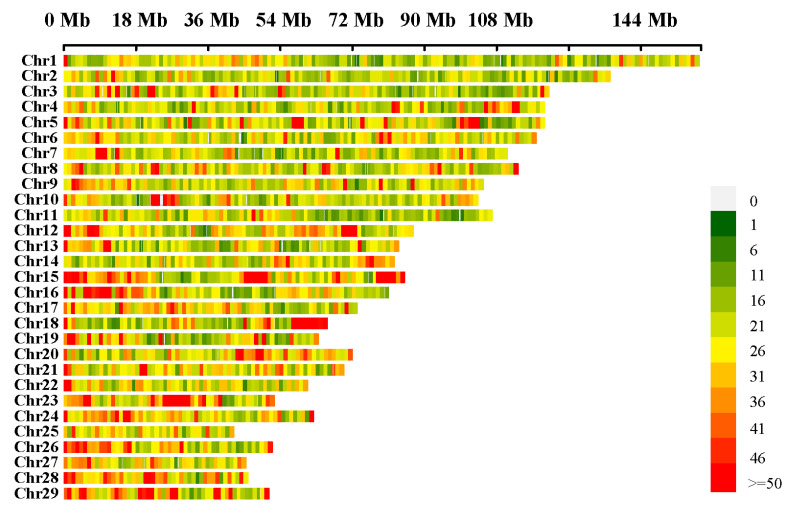
SV density map.

We measured 31 body conformation traits in 169 Xinjiang Brown cattle and retained 164 individuals for analysis after quality control. The standard deviation of these traits ranged from 0.47 (front teat diameter) to 10.96 (chest circumference), and the mean values of the 10 linear scoring traits ranged from 4.00 (front teat placement) to 6.30 (mid-rib shape). Descriptive statistics are listed in Supplementary Table S2. Following quality control of the corresponding SV dataset, we used 66,424 SVs for association analysis using the GCTA (v.1.94.1) software package (Figure 4). After genome-wide Bonferroni correction, 58 SVs were identified as significantly associated with five body conformation traits: chest width, rear udder width, udder depth, rump width, and heel depth. No significant SVs were detected for the remaining traits (Figure S2).

**Figure 5:**
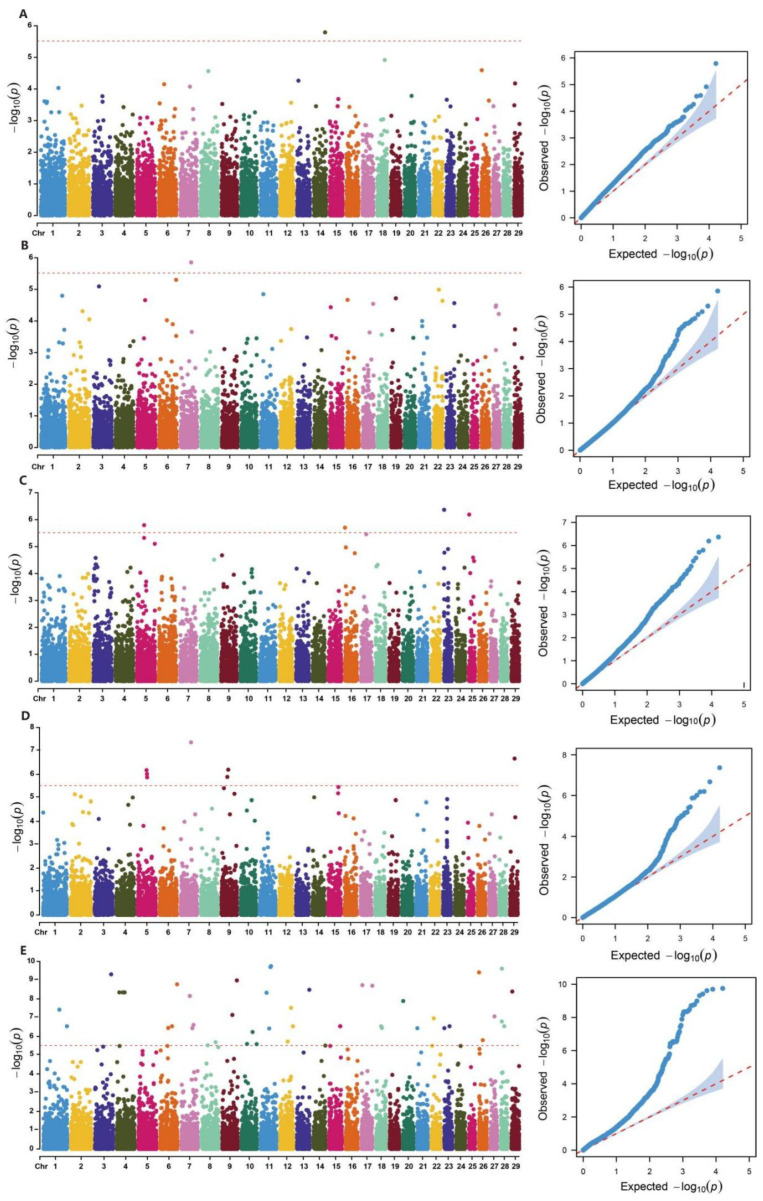
Manhattan and QQ plots for the association analysis of body conformation traits in Xinjiang Brown cattle. Note: The *x*-axis represents chromosomes, and the *y*-axis represents the −log₁₀ (*p*-value) of SVs. The red line indicates the genome-wide significance threshold (0.05/N). (**A**): chest width; (**B**): udder depth; (**C**): rear udder width; (**D**): rump width; (**E**): heel depth.

For chest width, a significant SV was detected on chromosome 14. For rear udder width, four significant SVs were detected on chromosomes 5, 16, 23, and 25. For udder depth, one significant SV was detected on chromosome 7. For rump width, seven significant SVs were detected on chromosomes 5, 7, 9, and 29. For heel depth, 45 significant SVs were detected across 22 chromosomes (Figure 5). Among the 58 genome-wide significant SVs, most were deletions, followed by duplications, inversions, and translocations. The lengths of these SVs varied widely from 0 and 132,056 bp. The functional annotation of these 58 significant SVs using ANNOVAR identified 67 genes (Supplementary Table S3).

**Figure 6:**
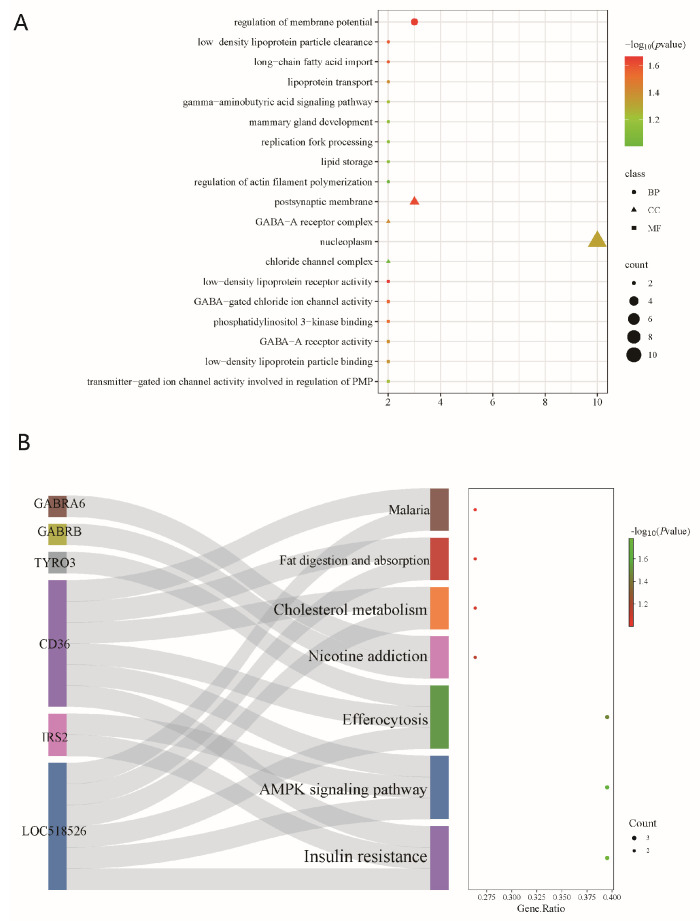
(**A**) GO and (**B**) KEGG enrichment analysis.

To further explore the biological functions of the candidate genes identified through SV association analysis, we performed GO and KEGG enrichment analyses on the 67 candidate genes identified via GWAS. GO analysis revealed significant associations with long-chain fatty acid import, spermatogenesis, lipoprotein transport, and mammary gland development (Figure 6A). KEGG pathway enrichment analysis revealed significant enrichment in the adipocytokine signaling pathway, AMPK signaling pathway, and cholesterol metabolism (Figure 6B).

Further literature review revealed *CLINT1*, *EBF1*, *PAM16*, and *GRIP1* as potential candidate genes influencing udder traits (udder depth and rear udder width). We identified *CFAP54* and *SLC22A16* as genes potentially affecting rump width and *DOK5*, *ETAA1*, and *IPMK* as genes potentially affecting heel depth.

## 3. Discussion

Detecting genomic SVs using high-throughput sequencing data has become a mainstream approach. Pindel, SVDetect, Delly, LUMPY, and Manta are among the various SV detection algorithms developed for high-throughput genomic sequencing. SVs frequently occur in repetitive regions of the genome; thus, accurately identifying them remains a key challenge in related studies. To enhance detection accuracy and reduce false positives, we employed SVs in the Xinjiang Brown cattle genome using three software tools (Delly, LUMPY, and Manta). The final SV dataset was obtained by intersecting the results from these three tools. Delly utilizes SVs using read-pairing, split-read, and read-depth algorithms [21]. Manta primarily relies on split-read and read-pairing methods [22], whereas LUMPY integrates read-pairing, split-read, read-depth algorithms, and additional prior knowledge [23]. Analysis of the autosomal distribution of these SVs revealed that chromosome 1 harbored the highest number of SVs. We observed a similar distribution pattern for SNPs [24] and CNVs [25]. Regarding SV types and frequencies, deletions constituted the main variation type, accounting for 48.21% of all variations, whereas insertions were the least common, comprising only 0.02%. We hypothesize that this pattern may result from two primary factors. First, the limitations of sequencing technology: compared with first- and third-generation sequencing, second-generation sequencing produces shorter reads, which may be insufficient to span entire insertion regions, thereby hindering accurate breakpoint identification. Second, the repetitive nature of genomic sequences where SVs typically occur further complicates detection. Pan-genomes integrate multiple genomic sequences, overcoming single-reference limitations to enhance SV detection accuracy. Declining sequencing costs and improved algorithms now enable better genetic analysis of key traits like disease resistance and growth rate.

Through gene annotation of high-frequency SVs, we identified 2016 protein-coding genes. The functional enrichment analysis of these genes revealed several pathways and associated genes linked to the superior traits of Xinjiang Brown cattle, including milk production, growth and development, and immune function. The *HDAC4* gene promotes muscle-derived cell growth by mediating soluble factors from muscle sources, thereby enhancing muscle cell proliferation and inhibiting differentiation [26]. Additionally, *HDAC4* can suppress the expression of myocyte enhancer factor 2 (*MEF2*), thus facilitating the differentiation of satellite cells in bovine skeletal muscle [27]. Moreover, we identified a deletion in an exon of the *MEF2A* gene, a member of the MADS transcription factor family that plays a crucial role in skeletal muscle development. Three SNPs in exon 11 of *MEF2A* influence early growth and body weight in Chinese indigenous cattle [28]. Furthermore, SNPs located in the 5′UTR, exon 4, and intron 7 of *MEF2A* are associated with carcass traits in chickens [29]. Therefore, these SVs could serve as candidate markers for the growth and development of Xinjiang Brown cattle.

Through years of selective breeding and genetic improvement, Xinjiang Brown cattle have developed superior dairy traits, including high milk yield and excellent milk quality. Here, we identified a deletion in the intron of the *TP63* gene, which regulates the growth and differentiation of mammary epithelial cells. It is also a marker for high milk production in dairy cattle [30]. Additionally, we detected a deletion in the intergenic region of the *FABP4* gene, which is associated with lipid metabolism and the expression of fatty acid-responsive genes. This gene is located on chromosome 14, within a QTL-rich region (46,833,665–46,838,053) [31,32] where QTLs related to milk yield have been identified.

Furthermore, we identified a deletion in the intron of the *PRKG1* gene on chromosome 26. This gene may affect fatty acid composition in Chinese Holstein cattle and significantly influences the synthesis of medium-chain saturated fatty acids in dairy cows [33]. Therefore, these SVs could serve as genetic markers for milk production traits. Xinjiang Brown cattle are also known for their strong disease resistance. Here, we identified a deletion in the intron of the *NFKBIZ* gene, which encodes the IκBζ protein, a key regulator in the pathogenesis of mastitis. *NFKBIZ* is strongly associated with Streptococcus infections in mastitis and is a potential genetic marker for mastitis resistance in dairy cattle [34]. Additionally, we found a deletion in the intron of the *PTPRT* gene, which is linked to resistance against Mycobacterium infections [35]. Moreover, functional enrichment analysis identified several immune-related pathways significantly associated with disease resistance, including antigen-stimulated inflammatory responses, positive regulation of interleukin-2 production, interleukin-1 receptor binding, and cellular responses to hormonal stimuli. These findings further support the strong immune resistance and disease tolerance observed in Xinjiang Brown cattle.

As an important type of genetic variation, genomic SVs are closely associated with economic traits and disease susceptibility in animals and plants. For example, in the Meishan pig genome, a 140 bp deletion in the *IDO2* gene and a duplication in the *CYP2J2* gene have been linked to reproductive performance [36]. Here, we identified four genes (*CLINT1*, *EBF1*, *PAM16*, and *GRIP1*) that may influence udder traits. Specifically, a 3396 bp SV on chromosome 7 was associated with udder depth and was annotated to two genes, *CLINT1* and *EBF1*. *EBF1* encodes a transcription factor that regulates B cell, neuronal, and adipocyte differentiation [37]. Meanwhile, *CLINT1* plays a crucial role in intracellular transport and vesicle formation encoding a protein that interacts with clathrin, adaptor protein AP-1, and phosphoinositides—key factors in intracellular transport and vesicle trafficking [38]. Mutation or abnormal expression of *CLINT1* may disrupt the normal transport processes in mammary epithelial cells, thereby interfering with mammary gland development. Additionally, this gene influences cell size and proliferation [39]. We identified *PAM16* and *GRIP1* as candidate genes associated with variation in rear udder width. *PAM16* is involved in sphingolipid metabolism, which is essential for cell structure, signaling, and regulation and plays a critical role in cell growth, differentiation, and apoptosis [40]. It is also involved in mitochondrial protein transport and localization [41], suggesting a potential role in mammary gland development through mitochondrial function regulation. Furthermore, rear udder width is a key trait affecting milk yield in dairy cows. *PAM16* is associated with milk yield and protein production in dairy cattle [42], indicating that it may be involved in udder development. Meanwhile, *GRIP1* functions as a steroid receptor coactivator involved in nuclear receptor-mediated transcriptional activation. It activates myogenin, which plays a role in myocyte differentiation [43], suggesting that *GRIP1* influences rear udder width by affecting the differentiation of mammary muscle tissues. Rump traits are strongly correlated with reproductive traits, indicating that these key morphological traits influence reproductive performance. In this study, all the genes that we identified as associated with rump width are linked by function to reproduction. For example, the spermatogenesis pathway clearly emerged from the GO enrichment analysis, with *CFAP54* and *SLC22A16* as candidate genes. *CFAP54* is related to cilia structure and function and is associated with litter size in Yunling Black goats [44]. *SLC22A16* encodes a transporter protein involved in the transport of organic cations across cell membranes and is linked to growth and development in Beijing ducks [45]. While no direct reports have linked these two genes to rump width, multiple genes and environmental factors influence body conformation traits in cattle, rather than by a single gene. Hoof depth is a critical trait in limb and hoof structure and is closely associated with hoof disease susceptibility.

Here, we identified three candidate genes related to hoof depth: *DOK5*, *ETAA1*, and *IPMK*. *DOK5* is a member of the DOK family; it is involved in multiple biological processes, particularly cell signaling and metabolic regulation. It can regulate osteoblast proliferation and differentiation via the Wnt/β-catenin signaling pathway [46]. *IPMK* (Inositol Polyphosphate Multikinase) is a multifunctional kinase with broad substrate specificity. Beyond its catalytic activity, it interacts with key signaling molecules, such as mTOR, AMPK, and LKB1; it mediates growth, stress response, and energy homeostasis [47]. Given its central role in mammalian signaling networks, *IPMK* may also influence hoof depth. Meanwhile, *ETAA1* has been associated with growth traits in sheep. Wijayanti et al. reported that *ETAA1* significantly influenced growth traits and was associated with body conformation traits in Lanzhou big-tail sheep, Hu sheep, and Tan sheep [48]. These findings strongly indicate that *ETAA1* and *IPMK* influence hoof depth, a hypothesis that warrants further functional validation. Our previous study focused on SNP-based GWAS, estimating heritability and genetic parameters using pedigree and genomic data, revealing moderate-to-high heritability for body conformation traits [49]. Building on our prior SNP-based analyses, this study’s novelty lies in its comprehensive SV map and SV-GWAS. Both these findings provide novel insights into the genetic basis of body conformation traits in Xinjiang Brown cattle.

In addition to the five traits—chest width, udder depth, rear udder width, rump width, and hoof depth—our GWAS did not identify significant associations between SVs and the remaining body conformation traits. This can be attributed to several factors: although SVs are widely present in the genome and significantly influence phenotypes, due to limitations in detection technology, they often exhibit lower coverage and frequency than SNPs, which hinders the identification of sufficient SV–trait associations. Finally, SV detection algorithms and analytical techniques are relatively new and are continuously evolving. SV detection is inherently more complex than SNP detection methods and may have a higher error rate, which could lead to failure in identifying phenotype-associated SVs in SV-GWAS or erroneously exclude true associations. Additionally, because SVs typically span larger genomic regions and often span multiple genes or regulatory elements, interpreting the relationship between variations and phenotypes may be more difficult in SV-GWAS. However, advancements in third-generation sequencing technologies and the development of pangenome references should substantially improve the accuracy of SV identification, potentially overcoming the current limitations of SV-GWAS to a certain extent.

## 4. Materials and Methods

### 4.1. Experimental Materials

We selected 169 lactating and healthy adult Xinjiang Brown cattle from the core breeding group of the Xinjiang Ili New Brown Breeding Farm (Tilihala Village, Almale Township, Xinyuan County, Ili, Xinjiang, China). We collected blood samples (10 mL) via caudal vein puncture using disposable vacuum blood collection tubes containing EDTA as an anticoagulant. We aliquoted the samples and preserved them on dry ice before transporting them to the laboratory for DNA extraction.

### 4.2. Genomic DNA Extraction

We extracted genomic DNA from Xinjiang Brown cattle through the phenol-chloroform method. We assessed the concentration and quality of the extracted DNA using a NanoDrop 2000 spectrophotometer (Thermo Fisher Scientific Inc., Carlsbad, CA, USA) and agarose gel electrophoresis, respectively. We then stored the extracted DNA at −80 °C until further analysis.

### 4.3. Genome Sequencing, Quality Control, and Read Alignment

Whole-genome resequencing was performed by BGI-Shenzhen using high-quality genomic DNA samples. They constructed a 150-bp paired-end library and performed sequencing on the DNBSEQ-T7 platform (MGI Tech Co., Ltd., Shenzhen, Guangdong, China).

To ensure the reliability of downstream data analysis, quality assessment of the sequencing data was performed using FastQC (https://www.bioinformatics.babraham.ac.uk/projects/fastqc/) (accessed on 14 August 2023), and parameters such as GC content distribution, sequence quality, sequence length distribution, and base quality scores were evaluated. Subsequently, sequencing reads were quality-filtered using fastp [50], where low-quality reads and adapter sequences were removed to obtain high-quality clean reads. The filtering parameters were as follows: fastp -i -I -o -O -w 4 -q 20 -n 2 -u 30. After quality control, the high-quality clean reads were aligned with the cattle reference genome (ARS-UCD1.2 Btau_5.0.1Y) using BWA-MEM [51], generating BAM files containing alignment results. The alignment parameters were as follows: -t 12 -M -R ‘@RG\tID:$i\tLB:$i\tPL:ILLUMINA\tSM:$i’. Next, SortSam and MarkDuplicates modules from PICARD (https://broadinstitute.github.io/picard/ accessed on 27 May 2025) were used to sort the alignment results and remove PCR duplicates, ensuring high-quality mapped reads. Finally, sequencing depth, alignment rate, and other statistical metrics were evaluated from the processed BAM files using Qualimap v2.3 [52].

### 4.4. Structural Variation Detection and Genotyping

We employed three software tools: Delly v1.1.6 [21]—optimized for high sensitivity in small-to-medium SVs (50 bp–10 kb) with precise breakpoint resolution, combining paired-end and split-read signals; Manta v1.6.0 [22]—specialized in mid-to-large SVs (200 bp–1 Mb), excelling in paired-end and split-read analysis for germline and somatic variants; and LUMPY v0.3.1 [23]—a probabilistic framework integrating multiple signals (discordant pairs, split-reads, and prior evidence) for complex SV discovery, particularly robust in low-coverage or heterogeneous samples. We used the default parameters for SV detection in all three tools. Among them, Manta and Delly perform SV genotyping directly, while LUMPY requires additional processing through SVTyper for genotyping. We based our SV detection workflow on BAM files generated from sequence alignment. Each piece of software independently identified SVs for each sample, and we subsequently filtered the results using SURVIVOR v1.0.7 [53]. We only retained SVs detected by at least two of the three software tools, using SURVIVOR merge with these parameters: SURVIVOR merge file 1000 2 1 1 0 30. This approach ensured the integration of a high-quality SV dataset for downstream analyses.

### 4.5. Variant Annotation

We performed SV annotation based on the cattle reference genome annotation file (ARS-UCD1.2 Btau_5.0.1Y). We built the annotation database using the retrieve_seq_fro-m_fasta.pl module in ANNOVAR v2016-02-01 [54]. We then carried out the functional annotation of each filtered SV using the table_annovar.pl module, identifying protein-coding gene sets associated with SVs.

### 4.6. Genome-Wide Association Studies

We selected 31 body conformation traits for GWAS, using measurement methods consistent with those previously reported by Zhang et al. [54]. We obtained the phenotypic data for this study from the Xinjiang Ili New Brown Breeding Farm’s official body conformation and appearance records of adult Xinjiang Brown cattle. The analyzed traits included 10 linear scoring traits (mid-rib shape, rear leg side view, bone quality, hoof angle, rear leg rear view, fore udder attachment, rear udder length, udder balance, front teat placement, and rear teat placement) and 21 measured traits (body height, body length, chest circumference, cannon bone circumference, rump height, chest depth, chest width, withers width, rear leg girth, rear leg height, rump length, rump width, rump angle, rear udder width, rear udder height, median suspensory ligament, udder depth, fore udder length, front teat length, front teat diameter, and heel depth).

For GWAS, we filtered out SVs with a minor allele frequency < 0.05. After quality control, we retained 169 Xinjiang Brown cattle and 66,424 high-quality biallelic SVs for GWAS. We performed GWAS using a mixed linear model association approach [49,55] in the GCTA software package. This model incorporates the genomic relationship matrix to account for population structure and relatedness, thereby reducing false positives. The model is formulated as:

y=a+bx+g+e

In this model, ***y*** represents the phenotypic value, ***a*** denotes the overall mean, ***b*** represents the additive effect of the candidate SV (fixed effect) being tested, ***x*** is the SV genotype indicator variable, encoded as 0, 1, or 2, ***g*** represents the polygenic effect (random effect), accounting for the cumulative effect of all SVs, captured by the genomic relationship matrix, and ***e*** denotes the residual error. To facilitate calculations, we estimated the genetic variance (***g***) using a null model (***y*** = ***a*** + ***g*** + ***e***), and then held this variance as a constant while testing associations between each SV and the traits. To control for false positives due to multiple testing, we applied the Bonferroni correction method to the GWAS results using a threshold of 0.05/N, where N represents the total number of SVs retained after quality control. After correction, we set the genome-wide significance threshold to 3.04E-6. Finally, we visualized the GWAS results as Manhattan plots and QQ (Quantile-Quantile) plots using the CMplot R package v4.5.1.

### 4.7. Functional Gene Enrichment Analysis

We performed functional enrichment analysis of genes annotated from high-frequency SVs using the Database for Annotation, Visualization, and Integrated Discovery (DAVID v2023q4) (https://david.ncifcrf.gov/). This analysis included enrichment analysis for the Kyoto Encyclopedia of Genes and Genomes (KEGG) pathways and Gene Ontology (GO) terms. We then visualized the enrichment results using the online bioinformatics tool MicroBioinformatics (http://www.bioinformatics.com.cn/).

## 5. Conclusions

Based on whole-genome resequencing data from 169 Xinjiang Brown cattle, we systematically analyzed the distribution characteristics of SVs and identified a set of candidate genes associated with milk production, growth, and immunity, including *NFKBIZ*, *PTPRT*, *TP63*, and *FABP4*. Through GWAS, we identified 58 significant SVs and mapped them to genes potentially associated with udder depth (*CLINT1* and *EBF1*), rear udder width (*PAM16* and *GRIP1*), rump width (*CFAP54* and *SLC22A16*), and hoof depth (*DOK5*, *ETAA1*, and *IPMK*). These findings provide novel insights into the genetic basis of body conformation traits in Xinjiang Brown cattle and offer valuable resources for genetic improvement and precision breeding strategies.

## Data Availability

The data and material used in this research are available from the corresponding author on request.

## Supplementary Materials

The following supporting information can be downloaded at: https://www.mdpi.com/article/10.3390/ijms26115234/s1.
