# Supplementary material for: Identification of Genomic Structural Variations in Xinjiang Brown Cattle by Deep Sequencing and Their Association with Body Conformation Traits
Source: Int J Mol Sci. 2025 May 29;26(11):5234. doi: 10.3390/ijms26115234 (PMC12154528; doi:10.3390/ijms26115234)
Supplement: Supplementary file 1 [file ijms-26-05234-s001.zip › ijms-3565674-supplementary.pdf]

Supplementary Material

1 Supplementary Figures and Tables

1.1 Supplementary Tables

Table S1 169 Xinjiang Brown Cattle Sequencing Statistics

ID	Total reads	Mapped reads	Mapping rate	Number of nucleobase	GC content	Average depth
09-24A	640 094 288	639 292 715	99.87%	95 254 455 633	43.24%	34.5354X
10-135A	617 567 049	617 042 783	99.92%	91 927 928 766	42.66%	33.3302X
10-40A	598 039 057	597 085 682	99.84%	88 989 617 338	42.86%	32.2646X
11-99A	637 256 812	636 537 249	99.89%	94 741 421 152	42.83%	34.3499X
12-55A	574 076 850	570 068 907	99.30%	84 855 540 468	43.02%	30.7655X
13-70A	581 261 791	579 839 705	99.76%	86 423 986 111	42.60%	31.3343X
14-105A	734 732 828	733 771 422	99.87%	109 076 996 920	42.69%	39.548X
14-112A	589 227 827	589 107 000	99.98%	87 819 173 420	41.35%	31.8393X
14-98A	504 072 043	503 438 209	99.87%	74 987 254 103	42.53%	27.1873X
15-110A	567 842 670	567 196 411	99.89%	84 556 164 303	42.67%	30.6569X
15-119A	651 690 814	650 860 268	99.87%	96 720 566 713	42.68%	35.0677X
15-37A	651 871 077	651 051 650	99.87%	96 757 014 183	42.55%	35.0816X
16-158A	558 560 336	557 693 534	99.84%	83 123 687 277	42.71%	30.1375X
16-32A	687 612 743	686 674 559	99.86%	102 073 463 282	42.68%	37.0088X
16-39A	553 860 543	553 292 965	99.90%	82 420 834 209	42.72%	29.8827X
16-55A	654 050 353	653 350 902	99.89%	97 323 542 860	42.69%	35.286X
16-61A	631 236 376	630 477 344	99.88%	93 962 593 139	42.68%	34.0673X
16-77A	666 338 728	665 531 549	99.88%	99 192 386 540	42.50%	35.9634X
16-80A	692 648 555	691 907 968	99.89%	103 042 327 674	42.82%	37.3593X
17-05A	558 338 021	557 540 371	99.86%	82 790 490 289	42.81%	30.0165X
17-08A	588 926 534	588 154 932	99.87%	87 605 804 201	42.65%	31.7629X
17-102A	643 426 869	641 848 074	99.75%	95 565 074 149	42.66%	34.6484X
17-141A	586 743 889	586 042 678	99.88%	87 339 473 440	42.74%	31.6661X
17-157A	933 465 494	932 356 909	99.88%	137 979 251 782	42.89%	50.0265X
17-176A	649 890 159	649 013 367	99.87%	96 390 246 110	42.80%	34.9501X
17-246A	787 363 974	786 383 424	99.88%	116 639 733 650	42.68%	42.2925X
17-271A	676 710 688	675 918 349	99.88%	100 623 125 738	42.74%	36.4821X
17-277A	721 007 594	720 173 642	99.88%	106 979 534 238	42.83%	38.7901X
17-288A	738 902 773	738 291 852	99.92%	109 778 955 240	42.62%	39.8023X
17-290A	685 315 711	684 571 123	99.89%	101 954 945 273	42.80%	36.9652X
17-92A	763 761 173	761 699 864	99.73%	113 348 007 527	42.79%	41.0956X
17-99A	764 272 870	763 455 030	99.89%	113 181 954 257	42.64%	41.0362X
18-08A	706 170 531	705 383 156	99.89%	104 828 871 938	42.79%	38.0081X

18-114A	709 501 874	708 830 217	99.91%	104 979 107 069	42.98%	38.062X
18-136A	706 526 237	706 401 245	99.98%	105 119 819 989	41.09%	38.1127X
18-154A	692 613 458	691 810 079	99.88%	102 854 613 201	42.55%	37.2922X
18-172A	650 133 208	649 383 486	99.88%	96 430 551 132	42.69%	34.965X
18-41A	661 467 728	660 703 335	99.88%	98 428 557 095	42.64%	35.6865X
18-58A	672 829 870	672 094 429	99.89%	99 934 523 176	42.71%	36.2333X
18-64A	808 916 243	807 600 428	99.84%	119 749 177 151	42.76%	43.4202X
18-76A	717 066 111	716 326 627	99.90%	106 132 216 104	42.60%	38.4799X
18-89A	654 101 264	653 373 523	99.89%	96 715 453 708	42.73%	35.0657X
18-91A	661 799 534	661 116 009	99.90%	98 230 977 216	42.78%	35.6174X
19-110A	681 700 896	680 806 365	99.87%	101 161 644 295	42.34%	36.678X
19-119A	667 062 659	666 404 983	99.90%	99 078 912 769	42.81%	35.923X
19-135A	700 425 735	699 753 665	99.90%	104 007 132 720	42.71%	37.712X
19-152A	746 995 487	746 318 278	99.91%	110 705 777 398	42.78%	40.1386X
19-166A	657 421 015	656 620 078	99.88%	97 351 068 638	42.67%	35.2967X
19-180A	728 223 276	727 144 912	99.85%	108 032 115 678	42.73%	39.1714X
19-236A	689 866 248	689 116 376	99.89%	102 266 634 528	43.04%	37.0805X
19-33A	722 522 359	721 702 331	99.89%	107 014 949 814	42.78%	38.8018X
19-44A	775 397 653	775 230 444	99.98%	114 806 987 460	42.02%	41.6247X
19-51A	875 066 092	874 382 624	99.92%	129 483 199 669	44.40%	46.9478X
19-68A	629 561 344	629 428 555	99.98%	93 748 095 147	41.06%	33.9896X
19-95A	966 289 775	965 106 131	99.88%	143 554 872 201	42.80%	52.048X
19-98A	614 923 467	614 776 741	99.98%	91 353 138 475	41.13%	33.1214X
65F103180156	397 694 749	397 315 743	99.90%	58 331 739 061	43.53%	21.1536X
65F103150160	341 563 531	341 214 013	99.90%	50 321 657 289	43.17%	18.249X
65F103190193	322 679 711	322 356 518	99.90%	47 744 572 785	43.31%	17.3143X
65F103170208	300 736 975	300 336 960	99.87%	44 342 907 894	43.15%	16.0808X
65F103170111	296 995 266	296 688 708	99.90%	43 892 968 074	43.35%	15.9176X
65F103190006	281 145 226	280 787 040	99.87%	41 180 495 186	43.37%	14.934X
65F103190204	275 062 728	274 818 591	99.91%	40 453 397 869	43.34%	14.6703X
65F103190032	258 913 664	258 524 943	99.85%	38 073 904 759	43.38%	13.8074X
65F103190188	250 288 120	250 041 157	99.90%	37 036 799 567	43.50%	13.4313X
65F103180131	237 158 042	236 869 277	99.88%	34 998 694 597	43.39%	12.6922X
65F103180044	234 666 557	234 450 397	99.91%	34 602 676 695	43.50%	12.5486X
65F103180082	229 937 669	229 680 772	99.89%	33 993 714 959	43.24%	12.3276X
65F103190177	227 931 860	227 649 514	99.88%	33 614 344 502	43.38%	12.1902X
65F103130042	222 308 920	222 061 839	99.89%	32 868 999 955	43.47%	11.9198X
65F103170153	224 268 048	224 069 820	99.91%	32 855 024 373	43.30%	11.9125X
65F103130057	220 298 141	220 057 491	99.89%	32 505 069 290	43.20%	11.7879X
65F103190276	220 964 833	220 707 995	99.88%	32 399 285 378	43.22%	11.7495X
65F103190130	214 704 091	214 488 430	99.90%	31 707 020 919	43.23%	11.4985X
65F103190185	210 840 567	210 653 714	99.91%	30 853 713 422	43.33%	11.1868X
65F103150150	207 656 955	207 424 400	99.89%	30 710 808 294	43.23%	11.1354X
65F103140028	207 418 164	207 180 305	99.89%	30 670 290 095	43.36%	11.1209X

65F103190153	208 233 818	207 838 627	99.81%	30 658 412 910	43.15%	11.1166X
65F103170066	208 724 047	208 500 259	99.89%	30 643 603 310	43.19%	11.1111X
65F103150084	207 375 944	207 093 100	99.86%	30 626 304 113	43.35%	11.1047X
65F103190189	206 755 335	206 553 268	99.90%	30 529 434 895	43.20%	11.0698X
65F103170093	207 427 572	207 271 166	99.92%	30 523 454 842	43.42%	11.0676X
65F103160048	206 743 612	206 554 789	99.91%	30 413 389 109	43.39%	11.0272X
65F103140040	206 456 681	206 246 417	99.90%	30 398 827 417	43.07%	11.0222X
65F103160082	207 506 248	207 272 237	99.89%	30 392 802 175	43.22%	11.0201X
65F103190194	207 524 580	207 300 962	99.89%	30 382 092 976	43.49%	11.0161X
65F103200133	205 625 302	205 370 480	99.88%	30 380 483 790	43.30%	11.0158X
65F103200193	205 054 932	204 835 011	99.89%	30 325 857 142	43.34%	10.9954X
65F103190053	205 934 133	205 792 992	99.93%	30 304 268 462	43.26%	10.988X
65F103170087	205 210 263	205 006 483	99.90%	30 281 160 780	43.29%	10.9799X
65F103180069	204 515 282	204 299 116	99.89%	30 250 640 797	43.21%	10.9685X
65F103200024	204 259 304	204 033 605	99.89%	30 207 630 530	43.36%	10.9529X
65F103190151	204 450 191	204 175 751	99.87%	30 160 725 935	43.17%	10.9377X
65F103150141	203 963 400	203 750 027	99.90%	30 148 974 177	43.20%	10.9315X
65F103180177	204 528 451	204 252 906	99.87%	30 140 094 503	43.51%	10.9299X
65F103180244	204 295 929	204 102 451	99.91%	30 107 827 154	43.32%	10.9164X
65F103180164	204 073 609	203 842 378	99.89%	30 093 993 219	43.44%	10.9117X
65F103150128	203 587 425	203 384 923	99.90%	30 087 433 775	43.25%	10.9094X
65F103190161	203 330 532	203 104 153	99.89%	30 067 939 856	43.26%	10.9023X
65F103160101	204 596 203	204 391 776	99.90%	30 066 222 977	43.27%	10.9015X
65F103170019	203 757 140	203 589 603	99.92%	30 052 852 291	43.51%	10.8966X
65F103190170	203 427 718	203 219 021	99.90%	30 021 904 485	43.26%	10.8855X
65F103160006	203 445 511	203 258 737	99.91%	30 002 993 714	43.30%	10.8788X
65F103190223	202 809 879	202 588 360	99.89%	30 000 761 327	43.34%	10.8778X
65F103180204	202 558 063	202 375 562	99.91%	29 915 186 529	43.37%	10.8467X
65F103180056	203 862 297	203 685 470	99.91%	29 911 178 576	43.44%	10.8452X
65F103170038	203 119 836	202 882 349	99.88%	29 903 094 617	43.42%	10.8427X
65F103170173	202 041 257	201 841 611	99.90%	29 819 297 217	43.40%	10.8119X
65F103190121	201 324 196	201 049 403	99.86%	29 766 383 754	43.35%	10.793X
65F103200020	200 788 155	200 549 702	99.88%	29 686 471 849	43.16%	10.7641X
65F103160057	201 093 965	200 881 094	99.89%	29 619 186 266	43.17%	10.74X
65F103190184	200 711 822	200 404 245	99.85%	29 561 751 281	43.37%	10.7203X
65F103170192	200 418 427	200 068 359	99.83%	29 464 933 623	43.38%	10.6847X
65F103170167	198 899 505	198 664 166	99.88%	29 423 039 748	43.41%	10.6686X
65F103190030	199 144 229	198 853 457	99.85%	29 378 023 877	43.15%	10.6534X
65F103190181	198 316 350	198 115 857	99.90%	29 359 973 912	43.34%	10.6452X
65F103170285	198 651 721	198 390 132	99.87%	29 347 844 710	43.40%	10.6428X
65F103170134	198 555 287	198 297 033	99.87%	29 334 478 156	43.35%	10.638X
65F103180129	198 972 000	198 741 257	99.88%	29 323 402 961	43.19%	10.6341X
65F103180174	198 039 211	197 793 042	99.88%	29 227 603 815	43.21%	10.5991X
65F103180179	198 330 330	198 145 100	99.91%	29 226 290 146	43.08%	10.5973X

65F103180051	198 472 129	198 356 389	99.94%	29 070 271 681	43.53%	10.5402X
65F103180205	197 991 668	197 809 371	99.91%	29 043 227 622	43.32%	10.5308X
65F103160176	197 079 327	196 743 491	99.83%	29 035 559 856	43.28%	10.529X
65F103190072	196 910 156	196 603 862	99.84%	29 035 383 901	43.36%	10.5293X
65F103190246	197 625 184	197 414 558	99.89%	28 990 227 212	43%	10.5112X
65F103180107	195 988 399	195 767 482	99.89%	28 945 428 769	43.34%	10.4956X
65F103190129	196 330 187	196 109 171	99.89%	28 932 415 800	43.29%	10.4922X
65F103190031	196 439 483	196 187 493	99.87%	28 930 602 573	43.24%	10.4915X
65F103190126	195 899 300	195 617 249	99.86%	28 795 890 953	43.66%	10.4423X
65F103140032	193 607 967	193 334 534	99.86%	28 573 893 180	43.50%	10.3616X
65F103180005	193 880 777	193 766 818	99.94%	28 488 446 126	43.50%	10.3293X
65F103150153	191 821 404	191 571 204	99.87%	28 361 805 836	42.98%	10.2839X
65F103190084	191 666 308	191 470 460	99.90%	28 307 640 718	43.19%	10.2637X
65F103160178	191 463 353	191 167 985	99.85%	28 198 760 502	43.37%	10.2257X
65F103190094	191 089 717	190 871 713	99.89%	28 193 948 742	42.95%	10.2244X
65F103190222	190 454 295	190 190 580	99.86%	28 103 587 359	43.39%	10.1911X
65F103160019	190 103 123	189 906 856	99.90%	28 066 963 497	43.26%	10.177X
65F103170229	190 756 561	190 557 603	99.90%	27 953 706 728	43.32%	10.1374X
65F103150149	188 290 145	188 092 467	99.90%	27 889 611 802	43.42%	10.1128X
65F103180112	188 960 821	188 713 718	99.87%	27 840 123 507	43.35%	10.0961X
65F103190117	186 897 716	186 670 162	99.88%	27 580 556 401	43.26%	10.0016X
09-142A	633 186 014	632 518 362	99.89%	94 065 488 170	42.86%	34.1049X
11-23A	707 842 759	706 392 631	99.80%	105 202 579 648	43.43%	38.1421X
11-30A	1 036 081 621	1 034 998 099	99.90%	154 003 431 274	42.18%	55.8363X
11-77A	648 741 318	647 986 073	99.88%	96 553 793 430	43.63%	35.0064X
12-43A	660 153 847	659 348 696	99.88%	98 131 031 065	42.60%	35.5787X
13-03A	689 332 580	688 495 766	99.88%	102 508 728 222	42.40%	37.1657X
13-10A	686 938 631	686 142 767	99.88%	102 045 890 248	42.89%	36.9981X
13-75A	717 454 846	716 051 218	99.80%	106 627 090 487	42.74%	38.6592X
14-52A	451 914 486	451 342 240	99.87%	67 183 385 118	42.39%	24.3583X
15-63A	631 678 293	631 081 238	99.91%	93 848 612 774	42.55%	34.026X
16-85A	762 252 912	761 535 285	99.91%	113 295 787 515	42.48%	41.0769X
17-166A	684 923 769	683 702 155	99.82%	101 847 514 803	42.59%	36.9257X
17-233A	690 107 887	689 603 679	99.93%	102 676 833 656	42.69%	37.2267X
18-139A	676 104 472	675 278 415	99.88%	100 526 280 403	43.09%	36.4468X
18-143A	635 586 367	634 815 195	99.88%	94 488 938 978	43.04%	34.2583X
18-152A	653 887 789	653 041 718	99.87%	97 163 718 766	42.70%	35.2277X
18-159A	691 722 850	690 945 821	99.89%	102 830 952 440	43.25%	37.2825X
18-160A	700 101 546	699 314 445	99.89%	104 020 569 013	42.50%	37.7139X
18-162A	610 391 180	609 873 766	99.92%	90 817 447 616	43.74%	32.9269X
18-206A	798 325 961	797 309 862	99.87%	118 766 761 520	42.49%	43.0604X
19-219A	632 490 575	631 803 878	99.89%	94 012 370 339	42.57%	34.0853X
19-26A	741 150 956	740 212 337	99.87%	110 238 046 138	42.56%	39.9683X

19-271A	603 821 345	602 978 984	99.86%	89 783 476 654	42.83%	32.5518X
19-41A	855 777 973	854 553 687	99.86%	127 284 215 273	42.98%	46.1481X
19-61A	783 807 817	782 939 325	99.89%	116 552 541 575	42.87%	42.2575X
20-51A	591 413 836	590 675 290	99.88%	87 969 833 071	42.51%	31.8945X
15-111A	159 163 293	158 912 364	99.84%	23 776 175 368	43.32%	8.6205X

Table S2 Descriptive statistics of body size traits in Xinjiang brown cattle

Traits	Minimum	Maximum	Mean	Standard deviation
Body height（BH）/cm	154	123	140.27	6.04
Rump length（RL）/cm	203	144	172.58	10.04
Chest girth（CG）/cm	227	176	200.54	10.96
Hoop Circumference（HC）/cm	27	19	22.07	1.14
Cross high（CH）/cm	155	123	141.03	5.76
Body depth（BD）/cm	91	68	79.80	5.01
Chest width（CW）/cm	34	20	25.91	3.18
Rump Width（RW）/cm	25	12	16.74	2.42
Hind leg half circumference（HLC）/cm	50	33	40.95	2.86
Hind leg height（HLH）/cm	88	64	76.23	4.81
Rump length（RL）/cm	60	46	53.41	2.71
Rump width（RW）/cm	27	11	21.85	2.28
Rump angle（RA）/cm	13	-5	3.99	3.23
Rear udder width（RUW）/cm	19	7	12.32	1.78
Rear udder Height （RUH）/cm	34	17	25.96	3.54
Suspensory ligament（SL）/cm	7	0	2.79	1.24
Udder depth（UD）/cm	20	-10	8.40	4.19
Anterior mammary area length（AAL）/cm	29	10	19.10	3.61
Anterior teat length（ATL）/cm	10	2	5.06	1.45
Anterior teat diameter/cm（ATD）/cm	4	2	2.69	0.47
Heel depth（HD）/cm	6.5	2.5	3.50	0.61
Rib and bone（RB）/	9	4	6.30	1.01
Rear legs side view（RLSV）	8	3	5.20	0.93
Bone quality（BQ）	8	4	6.21	0.67
Foot angle（FA）	7	3	4.66	0.79
Rear legs rear view（RLRV）	7	3	5.77	1.02
Fore udder attachment（FUA）	9	2	5.60	1.49
Rear udder length（RUL）	8	3	5.12	1.28
Udder balance（UB）	7	2	4.90	0.82
Fore teat placement（FTP）	6	2	4.00	1.06
Rear teat placement（RTP）	8	2	5.35	1.10

Table S3 Information on significant SVs for body size traits in Xinjiang brown cattle

SV id	Chromo-some	Start	End	Length	Type	Gene	Trait
DEL00089553	14	75737492	75746075	8583	DEL	LOC112449559	CW
DUP00043412	7	69555527	69589492	33965	DUP	CLINT1、EBF1	UD
BND00136275	23	901713	33852877	0	TRA	-	RUW
DEL00148350	25	3507270	3507346	76	DEL	PAM16	RUW
INV00027807	5	47087405	47089166	1761	INV	GRIP1	RUW
DUP00098235	16	50740	53943	3203	DUP	NONE、OR5L1	RUW
DEL00043397	7	69078517	69078787	270	DEL	CYFIP2,NIPAL4	RW
BND00167680	29	30559026	25246228	0	TRA	-	RW
DEL00055784	9	45662614	45662675	61	DEL	HACE1、TRNAC-ACA	RW
DEL00028349	5	57926041	57926096	55	DEL	LOC782591	RW
DEL00028682	5	60922093	60923397	1304	DEL	CFAP54	RW
MantaDEL:64879:0:0:0:0:0	9	39775579	39775722	143	DEL	SLC22A16	RW
DEL00028714	5	61852773	61854435	1662	DEL	MIR135A-2、TRNAD-GUC	RW
DEL00070334	11	69405905	69411151	5246	DEL	LCLAT1	HD
DEL00070191	11	65537612	65537668	56	DEL	LOC100139826、ETAA1	HD
DEL00160705	28	6631602	6631639	49	DEL	TRNAC-ACA、SLC35F3	HD
DEL00151423	26	891045	892653	1608	DEL	IPMK、ZWINT	HD
DEL00017927	3	107705069	107705151	82	DEL	TRNAG-CCC、POU3F1	HD
DEL00058918	9	98239755	98245145	5390	DEL	PACRG	HD
DEL00039144	6	113432225	113432260	35	DEL	TBC1D14	HD
DEL00104750	17	5954071	5954226	155	DEL	TRNAW-CCA、GATB	HD
DEL00109782	17	72231132	72231171	83	DEL	PI4KA	HD
DEL00084684	13	82151974	82152433	459	DEL	DOK5、TRNAC-GCA	HD
DEL00164996	29	5351441	5356967	5526	DEL	LOC112444837、LOC616911	HD
DEL00019528	4	16767789	16768143	354	DEL	NXPH1	HD

DUP00020683	4	40373979	40376409	2430	DUP	LOC518526、CD36	HD
DUP00021204	4	52208240	52209212	972	DUP	TES	HD
DEL00068525	11	39331012	39331395	383	DEL	CCDC85A、TRNAY-AUA	HD
MantaDEL:513 75:0:0:0:0:0	7	56397783	56397902	119	DEL	KCTD16、NONE	HD
DUP00120528	20	2319170	2348824	29654	DUP	KCNIP1	HD
DUP00078474	12	72190191	72289960	99769	DUP	LOC100337108、LOC112449109	HD
DUP00004567	1	105952 220	105952744	524	DUP	OTOL1、SPTSSB	HD
DUP00057192	9	67919330	67922822	3492	DUP	ARHGAP18、TMEM244	HD
DEL00158477	27	27350639	27350674	35	DEL	WRN、LOC112444611	HD
MantaBND:58 639:0:1:1:0:0:0	22	14890964	76953698	0	TRA	-	HD
DEL00160708	28	6645944	6653737	7793	DEL	TRNAC-ACA、SLC35F3	HD
BND00044011	7	81095402	54031730	0	TRA		HD
DEL00006542	1	156483 613	156483653	40	DEL	KCNH8	HD
DUP00079420	12	84570255	84570640	385	DUP	MYO16、IRS2	HD
MantaDEL:983 41:0:0:0:0:0	15	72640785	72641071	286	DEL	NONE、API5	HD
DUP00096880	15	74385523	74406529	21006	DUP	ALX4	HD
BND00112072	18	33349753	116850218	0	TRA	-	HD
DUP00141256	23	35024586	35078173	53587	DUP	PRP-VII	HD
DUP00036993	6	79427511	79450068	22557	DUP	LOC112447087、TECRL	HD
BND00162242	28	22104962	84424922	0	TRA		HD
DEL00112337	18	38874603	38875198	595	DEL	TRNAL-UAA、PMFBP1	HD
MantaBND:50 195:0:1:0:0:0:1	6	54031723	81095409	0	TRA	-	HD
DUP00043571	7	73300776	73301269	493	DUP	GABRB2、GABRA6	HD
BND00136204	23	270207	39740604	0	TRA	-	HD
DUP00126228	21	54149	186205	132056	DUP	LOC101908149	HD
BND00069757	11	57135969	104123304	0	TRA	-	HD
DEL00064312	10	74144493	74144938	445	DEL	SNAPC1、SYT16	HD

DEL00153931	26	25637367	25637627	260	DEL	ECHS1、SORCS3	HD
DEL00075900	12	49570306	49570514	208	DEL	KLF12、TRNAY-AUA	HD
DUP00051525	8	94174669	94223189	48520	DUP	LOC789815	HD
DEL00062244	10	36988428	36988470	42	DEL	TYRO3、MGA	HD
DEL00065940	10	101138<br>071	101138255	184	DEL	FOXN3	HD
DUP00048428	8	41663923	41743206	79283	DUP	PUM3	HD

Note: DEL means deletion; DIP means duplication; TRA means translocation; INV means inversion; INS means insertion.

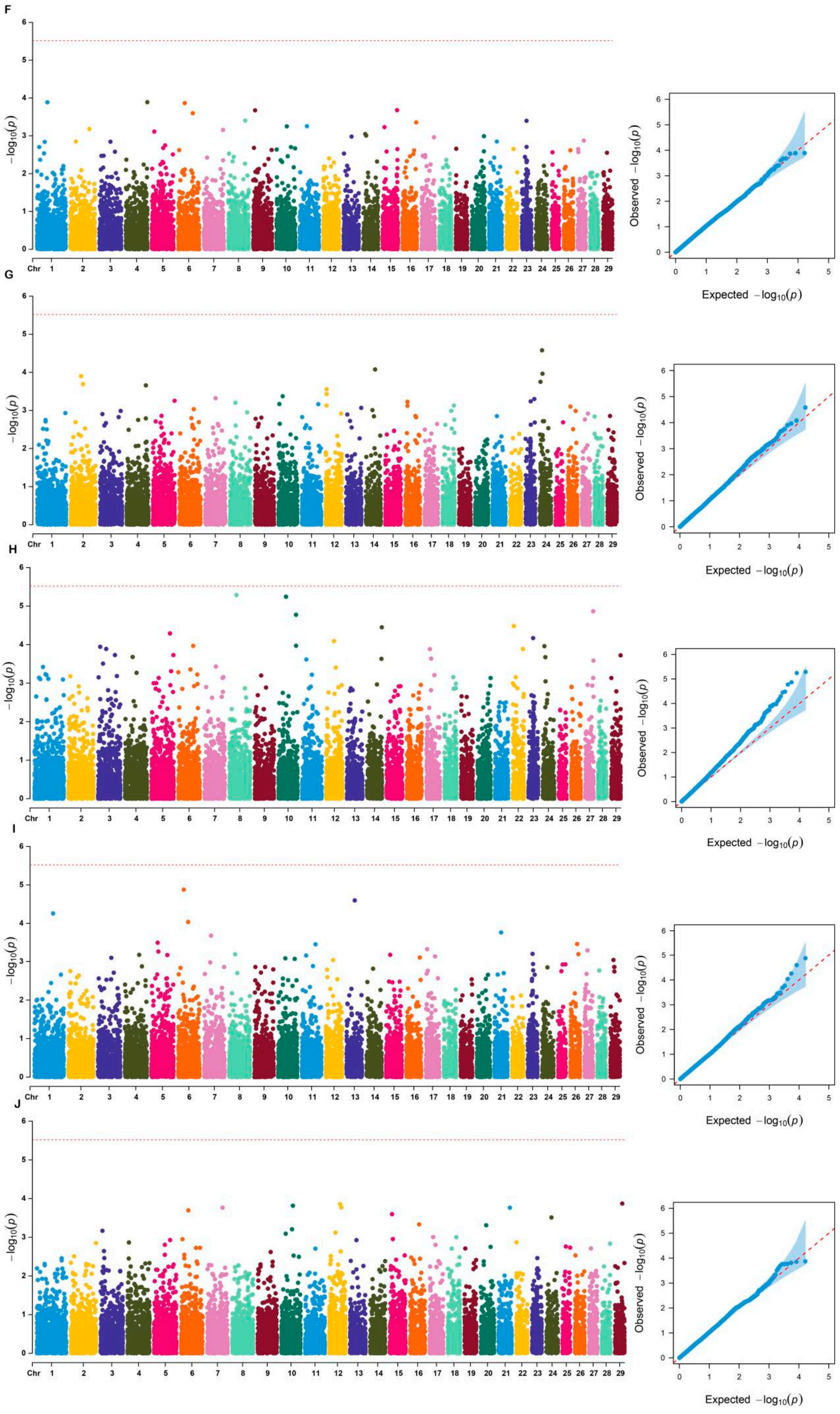

F
G
H
I
J
$-\log_{10}(p)$
Chr 1 2 3 4 5 6 7 8 9 10 11 12 13 14 15 16 17 18 19 20 21 22 23 24 25 26 27 28 29
Observed $-\log_{10}(p)$
Expected $-\log_{10}(p)$

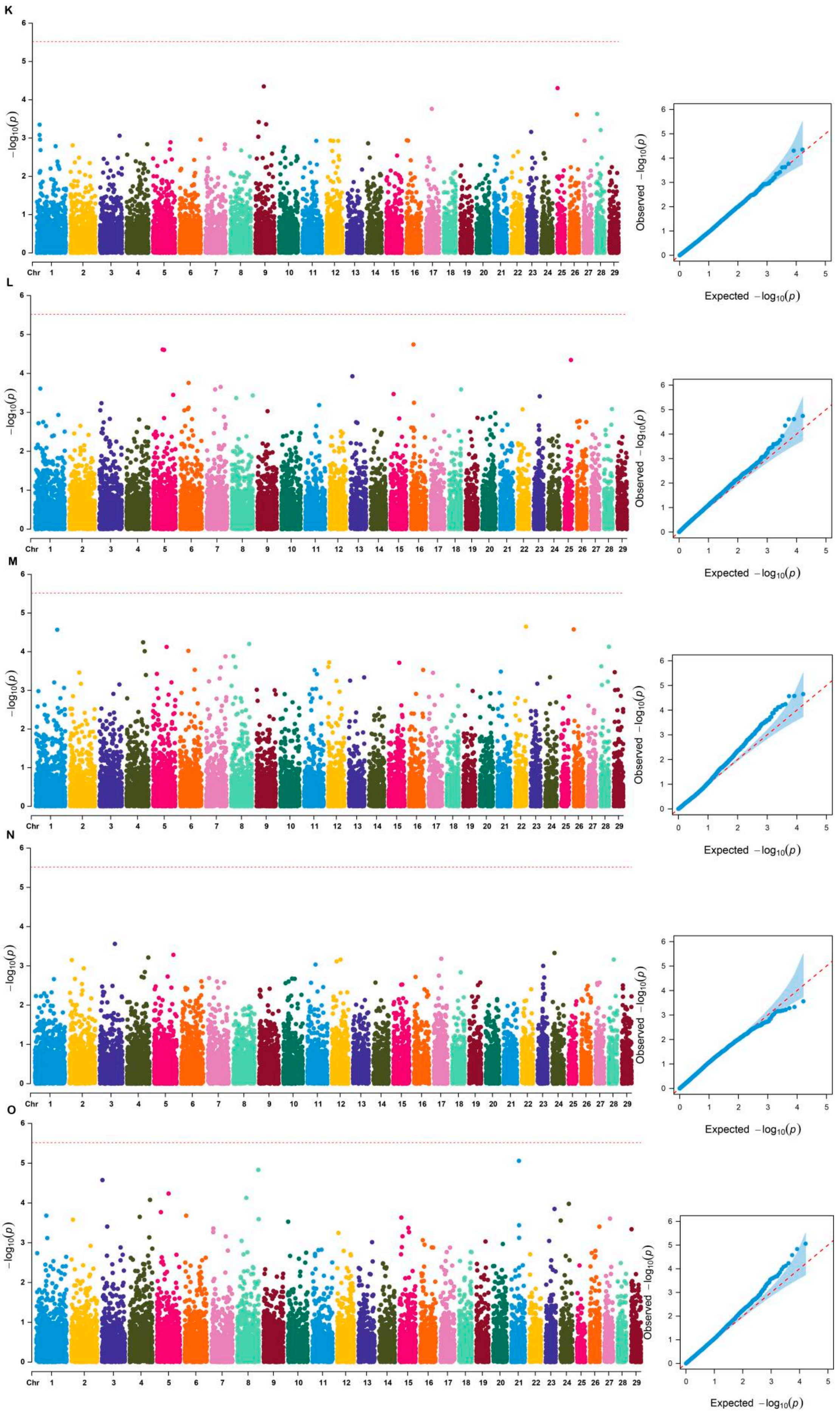
K
L
M
N
O
Chr
1 2 3 4 5 6 7 8 9 10 11 12 13 14 15 16 17 18 19 20 21 22 23 24 25 26 27 28 29
$-\log_{10}(p)$
Observed $-\log_{10}(p)$
Expected $-\log_{10}(p)$

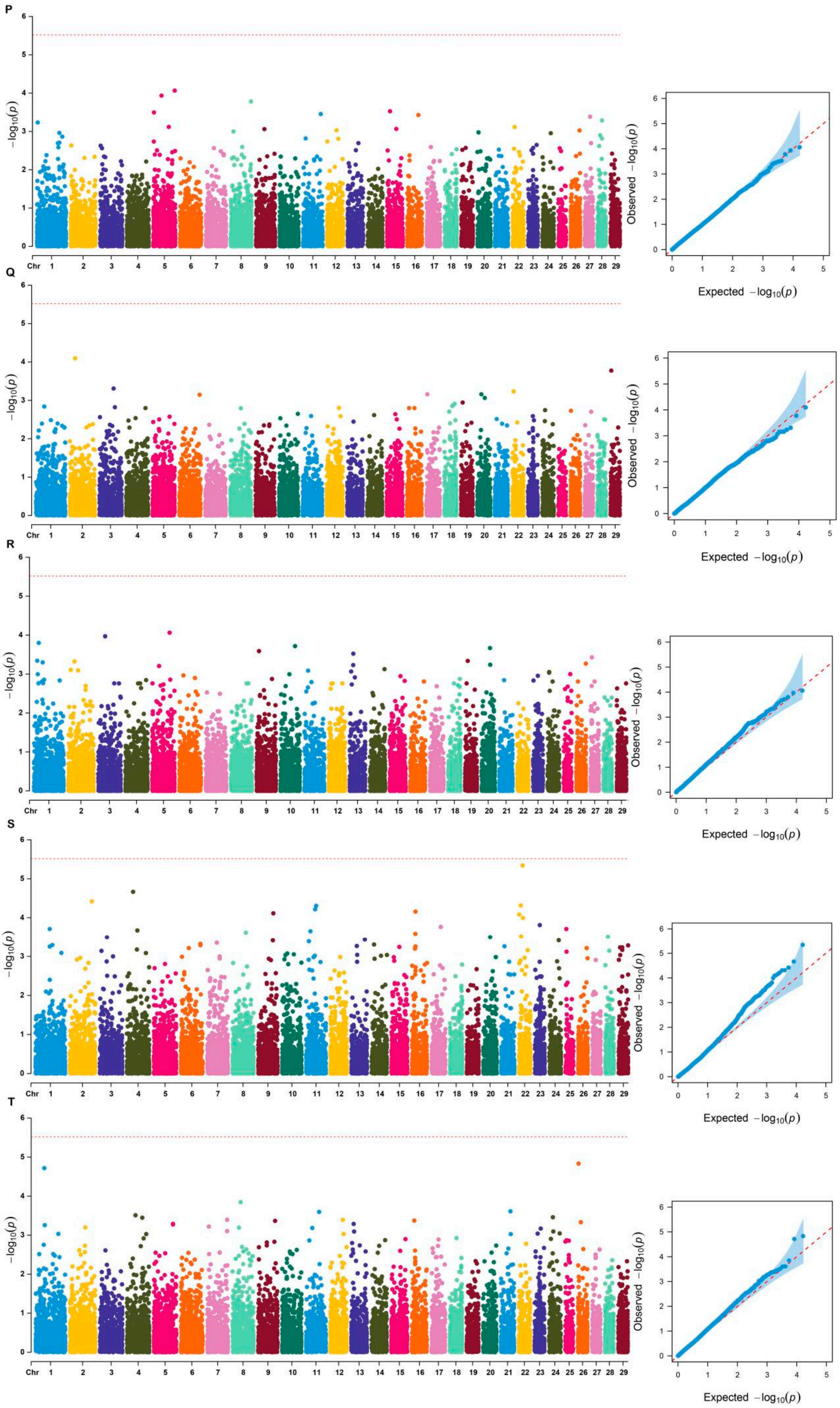
P
Q
R
S
T
−log10(p)
Chr 1 2 3 4 5 6 7 8 9 10 11 12 13 14 15 16 17 18 19 20 21 22 23 24 25 26 27 28 29
Observed −log10(p)
Expected −log10(p)

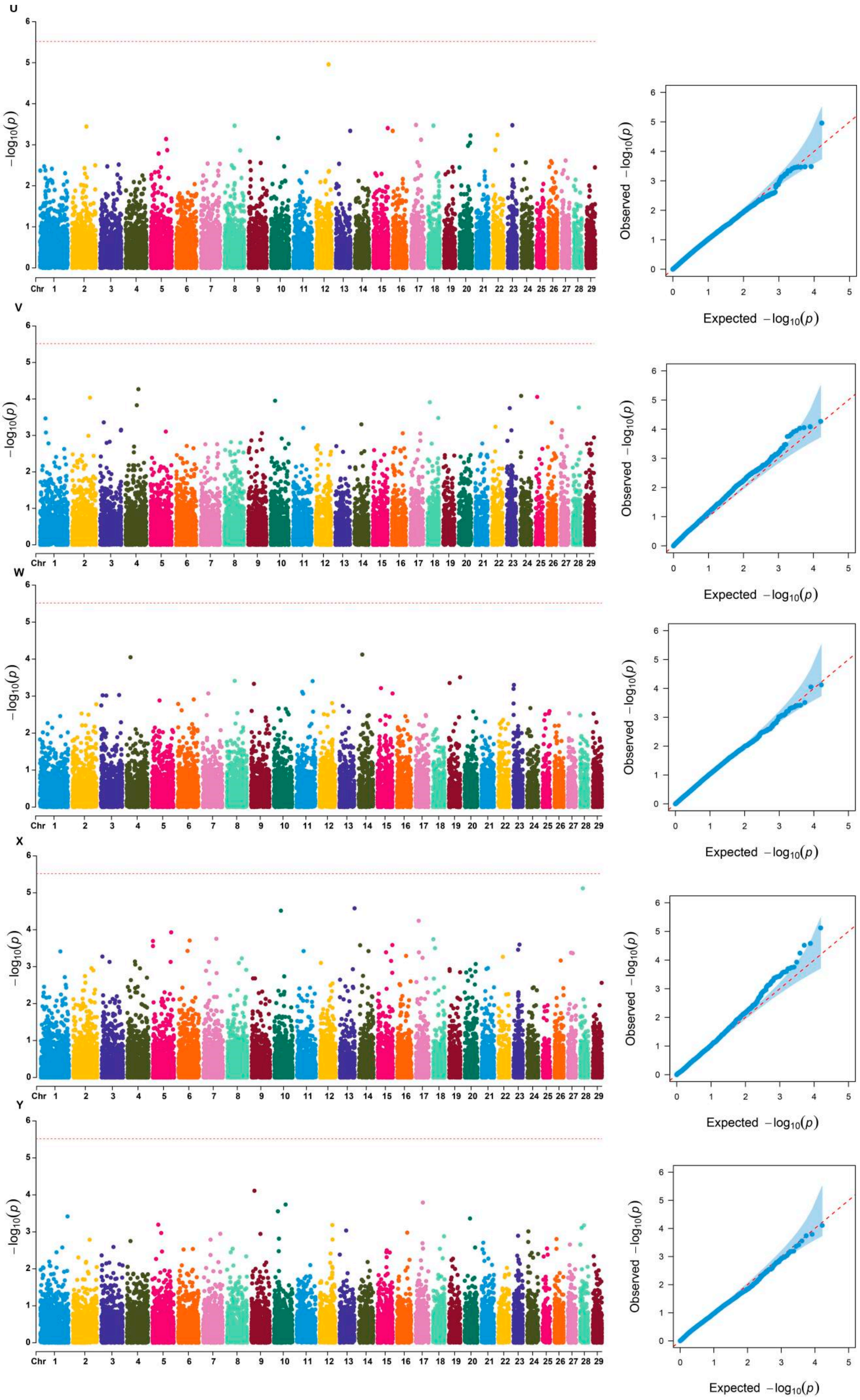
U
V
W
X
Y
−log10(p)
Chr 1 2 3 4 5 6 7 8 9 10 11 12 13 14 15 16 17 18 19 20 21 22 23 24 25 26 27 28 29
Observed −log10(p)
Expected −log10(p)

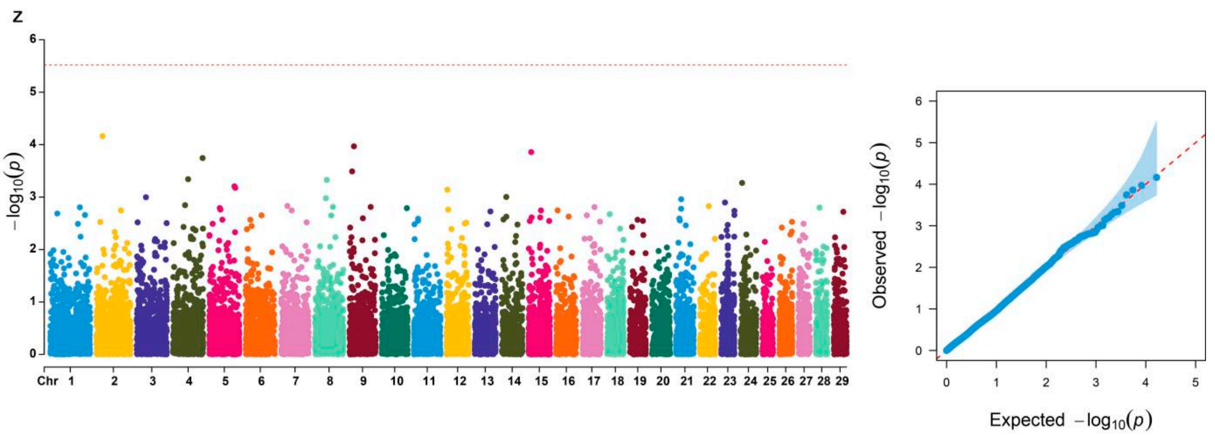

Figure S1 Genome-wide association analysis of body size traits in Xinjiang brown cattle Manhattan and QQ plots

Note：A-Z represent theBody height（BH）,Rump length（RL）,Chest girth（CG）,Hoop Circumference（HC）,Cross high（CH）,Body depth（BD）,Rump Width（RW）,Hind leg half circumference（HLC）,Hind leg height（HLH）,Rump length（RL）,Rump angle（RA）,Rear udder Height（RUH）,Suspensory ligament（SL）,Anterior mammary area length（AAL）,Anterior teat length（ATL）,Anterior teat diameter（ATD）,Heel depth（HD）,Rib and bone（RB）,Rear legs side view（RLSV）,Bone quality（BQ）,Foot angle（FA）,Rear legs rear view（RLRV）,Fore udder attachment（FUA）,Rear udder length（RUL）,Udder balance（UB）,Fore teat placement（FTP）,Rear teat placement（RTP）
